# Effect of vitamin D_3_ on the osteogenic differentiation of human periodontal ligament stromal cells under inflammatory conditions

**DOI:** 10.1111/jre.12858

**Published:** 2021-02-05

**Authors:** Alice Blufstein, Christian Behm, Barbara Kubin, Johannes Gahn, Xiaohui Rausch‐Fan, Andreas Moritz, Oleh Andrukhov

**Affiliations:** ^1^ Division of Conservative Dentistry and Periodontology University Clinic of Dentistry Medical University of Vienna Vienna Austria; ^2^ Division of Orthodontics University Clinic of Dentistry Medical University of Vienna Vienna Austria

**Keywords:** mesenchymal stromal cells, osteogenesis, periodontal ligament, vitamin D

## Abstract

**Objectives:**

Vitamin D_3_ is known to activate osteogenic differentiation of human periodontal ligament stromal cells (hPDLSCs). Recently, inflammatory stimuli were shown to inhibit the transcriptional activity of hPDLSCs, but their effect on vitamin D_3_‐induced osteogenic differentiation is not known. The present study aimed to investigate whether the effects of 1,25‐dihydroxvitamin D_3_ (1,25(OH)_2_D_3_) and 25‐hydroxvitamin D_3_ (25(OH)D_3_) on the osteogenic differentiation of hPDLSCs are also altered under inflammatory conditions. Furthermore, the expression of osteogenesis‐related factors by hPDLSCs under osteogenic conditions was assessed in the presence of inflammatory stimuli.

**Materials and Methods:**

Primary hPDLSCs of six donors were cultured in osteogenic induction medium containing either 1,25(OH)_2_D_3_ (0‐10 nM) or 25(OH)D_3_ (0‐100 nM) in the presence and absence of *Porphyromonas gingivalis* lipopolysaccharide (LPS) or Pam3CSK4 for 7, 14 and 21 days. Osteogenic differentiation of hPDLSCs was evaluated by analysis of mineralization as assessed by Alizarin Red S staining and gene expression levels of osteogenesis‐related factors osteocalcin, osteopontin and runt‐related transcription factor 2 (RUNX2) were analysed with qPCR.

**Results:**

Treatment with 1,25(OH)_2_D_3_ significantly enhanced the osteogenic differentiation of hPDLSCs and their expression of osteocalcin and osteopontin. The 1,25(OH)_2_D_3_‐triggered expression of osteogenesis‐related factors was significantly lower in the presence of Pam3CSK4, but not *P*.* gingivalis* LPS. None of the inflammatory stimuli had significant effects on the 1,25(OH)_2_D_3_‐induced osteogenic differentiation. 25(OH)D_3_ neither affected gene expression levels nor osteogenic differentiation of hPDLSCs cultured in osteogenic induction medium.

**Conclusion:**

The results of this study indicate that inflammatory stimuli also diminish the 1,25(OH)_2_D_3_‐induced expression of osteogenesis‐related factors in hPDLSCs under osteogenic conditions, while having no effect on the osteogenic differentiation.

## 1 INTRODUCTION

Vitamin D_3_ is a fat‐soluble steroid hormone that plays a pivotal role in numerous physiological functions, particularly bone metabolism.^1^ Although it can be obtained from several nutritional sources, humans are largely dependent on vitamin D_3_ production in the skin.^2^ Upon exposure to ultraviolet B radiation, 7‐dehydrocholesterol in the epidermis is photolysed to previtamin D_3_ and further converted to vitamin D_3_.^3^ This biologically inactive form is hydroxylated via the 25‐hydroxylase into the most abundant circulating vitamin D_3_ metabolite 25‐hydroxyvitamin D_3_ (25(OH)D_3_).^4^ The conversion into the biologically most active form 1,25‐dihydroxyvitamin D_3_ (1,25(OH)_2_D_3_) is facilitated via 1α‐hydroxylation.^5^ This process occurs predominantly in the kidneys, but can also be observed in extrarenal tissues, such as the periodontium.^5^, ^6^ 1,25(OH)_2_D_3_ exerts its numerous functions via binding to vitamin D receptor (VDR), which is expressed in almost all cells.^7^ Apart from its role in bone metabolism, the active vitamin D_3_ metabolite possesses strong immunomodulatory, anti‐inflammatory and anti‐proliferative properties.^8^, ^9^

Vitamin D_3_ has versatile effects in periodontal ligament stromal cells (hPDLSCs).^10^ hPDLSCs fulfill the minimal criteria of mesenchymal stromal cells and play a crucial role in periodontal tissue homeostasis.^11^, ^12^, ^13^ 1,25(OH)_2_D_3 _has been observed to enhance the osteogenic differentiation of hPDLSCs, to increase their expression of osteogenesis‐related factors and to dampen their inflammatory response.^6^, ^14^, ^15^

Considering these numerous positive effects, vitamin D_3_ deficiency is unsurprisingly associated with an increased risk of periodontal disease.^16^, ^17^, ^18^ Periodontitis is a multifactorial chronic disease leading to destruction of all periodontal tissues, namely gingiva, alveolar bone, cementum and periodontal ligament.^19^, ^20^ It is initiated by a shift of a symbiotic to a dysbiotic oral microbiota and is driven by an excessive inflammatory response.^21^

As summarized in our previous study, there are several reports about the influence of vitamin D_3_ supplementation during periodontitis treatment.^22^ Surprisingly, supplementation of vitamin D_3 _has never been shown to be beneficial during non‐surgical periodontal treatment so far.^22^ Our previous study focussed on finding a possible explanation for this. As inflammation is still strongly pronounced during the initial phase of periodontitis therapy, we investigated the effects of vitamin D_3_ metabolites on the expression of osteogenesis‐related factors in human periodontal ligament stromal cells (hPDLSCs) under inflammatory conditions. Inflammation was simulated by targeting Toll‐like receptors (TLRs), which recognize specific components of pathogens and lead to induction of inflammatory responses.^23^ TLR4 and TLR2 agonist *Porphyromonas gingivalis* (*P*.* gingivalis*) lipopolysaccharide (LPS) and TLR2/1 agonist Pam3CSK4 are known to strongly enhance the inflammatory response of hPDLSCs and were therefore chosen for the experiments.^24^, ^25^, ^26^ We observed that the 1,25(OH)_2_D_3_‐ and 25(OH)D_3_‐induced expression of osteogenesis‐related factors is diminished under inflammatory conditions, suggesting a decreased transcriptional activity of VDR in the presence of inflammatory stimuli.^22^

Since the gene expression of osteogenesis‐related factors does not always correlate with mineralization, it remained unclear whether inflammatory stimuli also affect the vitamin D_3_‐induced osteogenic differentiation.^27^ Therefore, the aim of the present study was to elucidate, if the 1,25(OH)_2_D_3_‐ and 25(OH)D_3_‐induced osteogenic differentiation of hPDLSCs is similarly affected by inflammatory conditions. In addition, the effects of inflammatory stimuli on the 1,25(OH)_2_D_3_‐ and 25(OH)D_3_‐triggered expression of osteogenesis‐related factors were assessed in hPDLSCs incubated in osteogenic induction medium.

It was hypothesized that the 1,25(OH)_2_D_3_‐ and 25(OH)D_3_‐induced osteogenic differentiation of hPDLSCs and the expression of osteogenesis‐related factors under osteogenic conditions are altered in the presence of inflammatory stimuli.

## 2 MATERIALS AND METHODS

### 2.1 Cell culture

Primary hPDLSCs were obtained from five periodontally healthy individuals undergoing third molar extraction due to orthodontic indications. The three female and two male donors were between 18 and 22 years old and had no chronic diseases or regular medication. Immediately after extraction, the teeth were gently washed with phosphate‐buffered saline (PBS) and the periodontal ligament tissue adhering to the middle third of the root was scraped off using a scalpel. The tissue fragments were incubated in Petri dishes containing Dulbecco´s modified Eagle´s medium (DMEM; Sigma‐Aldrich, St. Louis, USA) supplemented with 1% penicillin/streptomycin (P/S; Gibco, Carlsbad, USA) and 10% foetal bovine serum (FBS; Gibco, Carlsbad, USA) under humidified conditions at 5% CO_2_ and 37°C. After outgrowth of the hPDLSCs, they were transferred into cell culture flasks. Mesenchymal stromal cell character was assessed by flow cytometry analysis of characteristic mesenchymal surface markers CD29, CD90, CD105 and CD146, and negative expression of hematopoietic surface markers CD14, CD31, CD34 and CD45.^11^ Notably, the isolated cell population is heterogeneous and might additionally contain osteoblasts, fibroblasts and odontoblasts, which express similar surface markers as MSCs.^28^ However, the isolated cells still meet the minimal criteria of mesenchymal stromal cells as defined by the position papers of the International Society for Cellular Therapy.^11^, ^29^ Each of the subsequent experiments was conducted with hPDLSCs of five different donors within passage 4‐6.

### 2.2 Treatment protocol

hPDLSCs were seeded in 24 well plates at a density of 5 × 10^4^ cells/well together with 0.5 ml DMEM supplemented with 1% P/S and 10% FBS for 24 hours. For the following stimulation, osteogenic induction medium was prepared, which was composed of Minimum Essential Medium Eagle with alpha modification (α‐MEM; Sigma‐Aldrich, St. Louis, USA) supplemented with 20% FBS, 100 nM dexamethasone, 10 nM β‐glycerol phosphate and 0.05 nM ascorbic acid. After washing the hPDLSCs with PBS, cells were stimulated with osteogenic induction medium containing 1,25(OH)_2_D_3_ (0‐10 nM; Cayman Chemical, Ann Arbor, USA) or 25(OH)D_3_ (0‐100 nM; Cayman Chemical, Ann Arbor, USA) in the presence and absence of either TLR4 and TLR2 agonist standard *P*.* gingivalis* LPS (1 µg/ml; Invivogen, San Diego, USA) or Pam3CSK4, which activates TLR2‐TLR1 heterodimer (1 µg/ml; Invivogen, San Diego, USA). Since hPDLSCs lack of membrane‐bound CD14, LPS treatment was performed in the presence of soluble CD14 (0.25 µg/ml; Sigma‐Aldrich, St. Louis, USA) as reported previously, in order to enhance the LPS‐induced response.^25^ hPDLSCs incubated only with osteogenic induction medium served as control group. Every 72 hours, 0.25 ml of the stimulation media were replaced by freshly prepared ones. The total treatment was conducted for 7, 14 and 21 days. Each experiment was performed in duplicates for every donor.

### 2.3 Gene expression analysis

Quantitative polymerase chain reaction (qPCR) was performed in order to analyse the gene expression levels of osteogenesis‐related factors osteocalcin (BGLAP), osteopontin (SPP1) and runt‐related transcription factor 2 (RUNX2) in hPDLSCs treated for 7, 14 and 21 days. Cell lysis, mRNA extraction, transcription into cDNA and qPCR were executed with the TaqMan Gene Expression Cells‐to‐CT kit (Applied Biosystems, Foster City, USA). The Primus 96 advanced thermocycler (PeqLab/VWR, Darmstadt, Germany) was utilized to carry out reverse transcription. qPCR was performed with ABI StepOnePlus (Applied Biosystems, Foster City, USA) using following TaqMan Gene Expression Assays: BGLAP, Hs01587814_g1; SPP1, Hs00959010_,m1; RUNX2, Hs00231692_m1. The analysis was conducted in duplicates at 95°C for 10 minutes, 40 cycles, each for 15 seconds at 95°C and at 60°C for 1 minute. Quantification of the gene expression levels was executed with the 2^−ΔΔCt^ method applying following formula:

$$\Delta \Delta C_{t}\;={\left(C_{t}^{\text{target}}‐C_{t}^{\text{GAPDH}}\right)}_{\text{sample}}\;‐{\left(C_{t}^{\text{target}}‐C_{t}^{\text{GAPDH}}\right)}_{\text{control}}$$

hPDLSCs treated with osteogenic medium alone were considered as control group and Glyceraldehyde 3‐phosphate dehydrogenase (GAPDH) served as endogenous control.

### 2.4 Alizarin Red S staining

The presence of calcified deposition was assessed in hPDLSCs treated for 21 days by staining with Alizarin Red S. In detail, hPDLSCs were incubated with 4% paraformaldehyde in PBS for 10 minutes. After washing with PBS, cells were stained with Alizarin Red S (1.34 mg/ml; Sigma‐Aldrich, St. Louis, USA) dissolved in distilled water for 15 minutes. The stained hPDLSCs were washed with PBS and captured photographically, followed by quantification of the Alizarin Red S staining intensity. For this purpose, cells were incubated with 10% acetic acid for 30 minutes. Subsequently, supernatants were collected and incubated at 85°C for 15 minutes before putting them on ice. The cooled down samples were centrifuged at 13,400 *g* for 15 minutes and the supernatants were again collected and mixed with 10% ammonium hydroxide. Alizarin Red S staining solutions were prepared to serve as standard. The absorbance was read with a microplate reader at 405 nm.

### 2.5 Statistical analyses

The statistical analyses were conducted with SPSS 24.0 (IBM). Data were analysed by Friedman test, followed by Wilcoxon test for pairwise comparison. All data are presented as mean ± SEM of five independent experiments with five different donors performed in triplicates.

## 3 RESULTS

### 3.1 1,25(OH)_2_D_3_–induced osteocalcin expression in hPDLSCs under osteogenic conditions is diminished in the presence of Pam3CSK4

**FIGURE 1:**
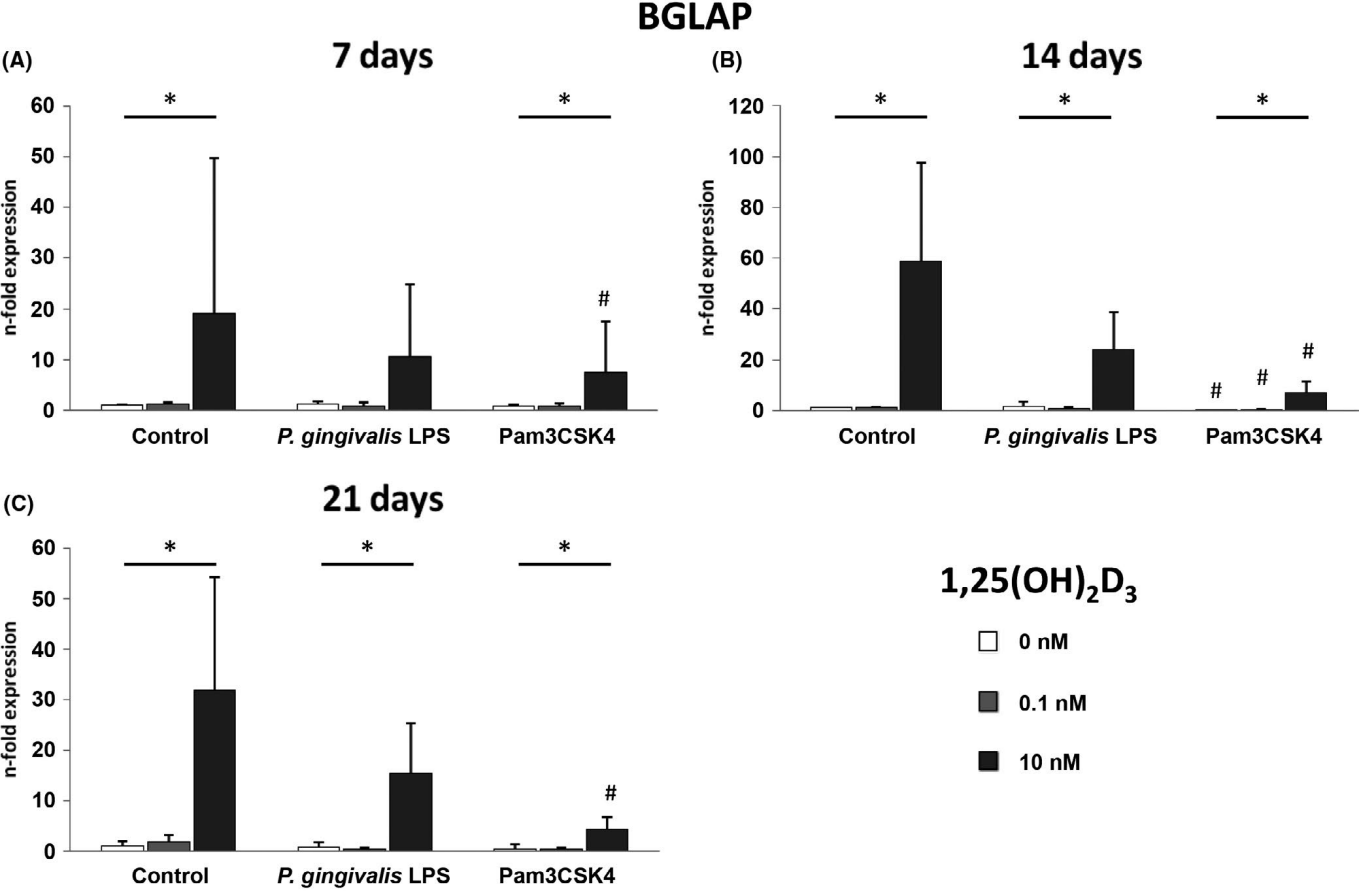
1,25(OH)_2_D_3_‐induced gene expression levels of osteocalcin under inflammatory and osteogenic conditions. Primary hPDLSCs (n = 5) were stimulated with osteogenic induction medium containing 1,25(OH)_2_D_3_ (0, 0.1, 10 nM) in the presence and absence of *P. gingivalis* LPS (1 µg/ml) or Pam3CSK4 (1 µg/ml) for 7 (A), 14 (B) or 21 (C) days. Resulting gene expression levels of osteocalcin were analysed with qPCR. Y‐axes represent the n‐fold expression of the target gene in relation with untreated hPDLSCs. Data are presented as mean ±standard error of the mean of five independent experiments. *Significant difference between groups, *p* < 0.05; # Significant decrease compared with respective vitamin D_3_ concentration in the absence of inflammatory stimuli, *p* < 0.05

Gene expression of osteocalcin by hPDLSCs after culture in osteogenic induction medium containing 1,25(OH)_2_D_3_ (0, 0.1, 10 nM) in the presence and absence of *P*. *gingivalis* LPS (1 µg/ml) or Pam3CSK4 (1 µg/ml) for 7 (A), 14 (B) and 21 (C) days is demonstrated in Figure 1. While 0.1 nM 1,25(OH)_2_D_3_ had no effect on the basal expression of osteocalcin under physiological conditions, it was significantly enhanced by the highest 1,25(OH)_2_D_3_ concentration after 7, 14 and 21 days.

In the presence of Pam3CSK4, the 10 nM 1,25(OH)_2_D_3_‐triggered osteocalcin expression was significantly decreased compared with physiological conditions at all tested time points. Interestingly, after 14 days incubation, treatment with Pam3CSK4 also led to a significant decrease of the basal and 0.1 nM 1,25(OH)_2_D_3_‐induced osteocalcin expression. Inflammatory conditions simulated with *P*.* gingivalis* LPS similarly diminished the osteocalcin expression induced by 10 nM 1,25(OH)_2_D_3_ after 7 (*P* = 0.116), 14 (*P* = 0.063) and 21 (*P* = 0.398) days, however, without a statistical significance.

### 3.2 Osteopontin gene expression levels of hPDLSCs induced by 1,25(OH)_2_D_3_ under osteogenic conditions are partially decreased by Pam3CSK4 and *P. gingivalis* LPS

**FIGURE 2:**
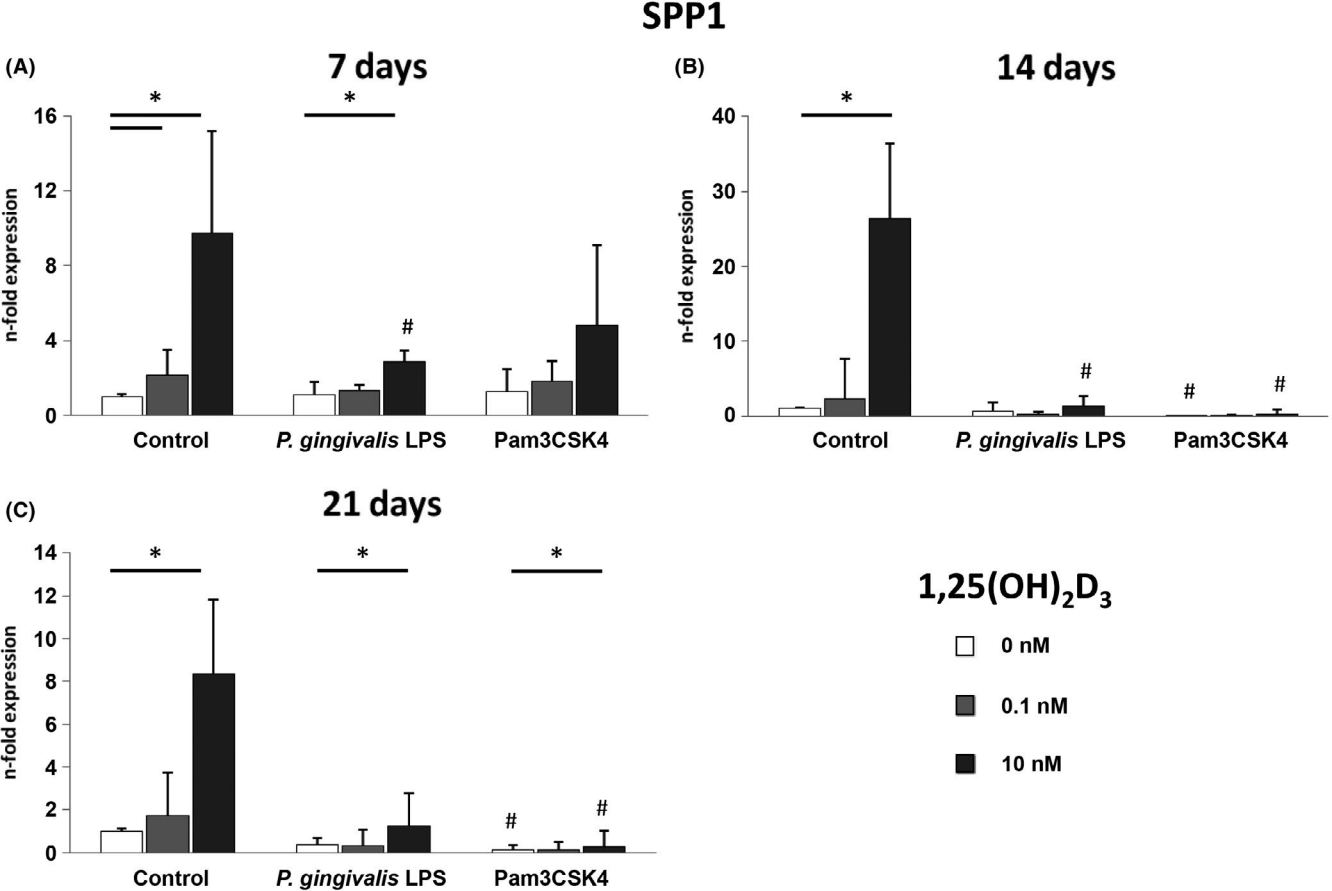
1,25(OH)_2_D_3_‐induced gene expression levels of osteopontin under inflammatory and osteogenic conditions. Primary hPDLSCs (n = 5) were stimulated with osteogenic induction medium containing 1,25(OH)_2_D_3_ (0, 0.1, 10 nM) in the presence and absence of *P. gingivalis* LPS (1 µg/ml) or Pam3CSK4 (1 µg/ml) for 7 (A), 14 (B) or 21 (C) days. Resulting gene expression levels of osteopontin were analysed with qPCR. Y‐axes represent the n‐fold expression of the target gene in relation to untreated hPDLSCs. Data are presented as mean ±standard error of the mean of five independent experiments. *Significant difference between groups, *p* < 0.05; # Significant decrease compared with respective vitamin D_3_ concentration in the absence of inflammatory stimuli, *p* < 0.05

Figure 2 illustrates the gene expression levels of osteopontin in hPDLSCs cultured in osteogenic induction medium supplemented with 0.1 or 10 nM 1,25(OH)_2_D_3_ under physiological and inflammatory conditions simulated with either *P*.* gingivalis* LPS (1 µg/ml) or Pam3CSK4 (1 µg/ml). Treatment of hPDLSCs was performed for 7 (A), 14 (B) and 21 (C) days. Similarly to osteocalcin, the expression of osteopontin was significantly enhanced by 10 nM 1,25(OH)_2_D_3_ in the absence of inflammatory stimuli after all treatment periods. In addition, a significant increase of the osteocalcin expression could be observed after treatment with 0.1 nM 1,25(OH)_2_D_3_ under physiological conditions for 7 days. The osteopontin expression induced by 10 nM 1,25(OH)_2_D_3_ was significantly diminished in the presence of *P*. *gingivalis* LPS after 7 and 14 days in comparison with physiological conditions. A similar tendency was observed after 21 days, but the differences were not statistically significant (*P* = 0.075). Pam3CSK4 had no significant effect on the 10 nM 1,25(OH)_2_D_3_‐triggered osteopontin expression after 7 days (*P* = 0.113), but led to a significant decrease of the osteopontin gene expression levels after 14 and 21 days.

### 3.3 1,25(OH)_2_D_3 _has no effect on the gene expression levels of RUNX2 in hPDLSCs under osteogenic and inflammatory conditions

**FIGURE 3:**
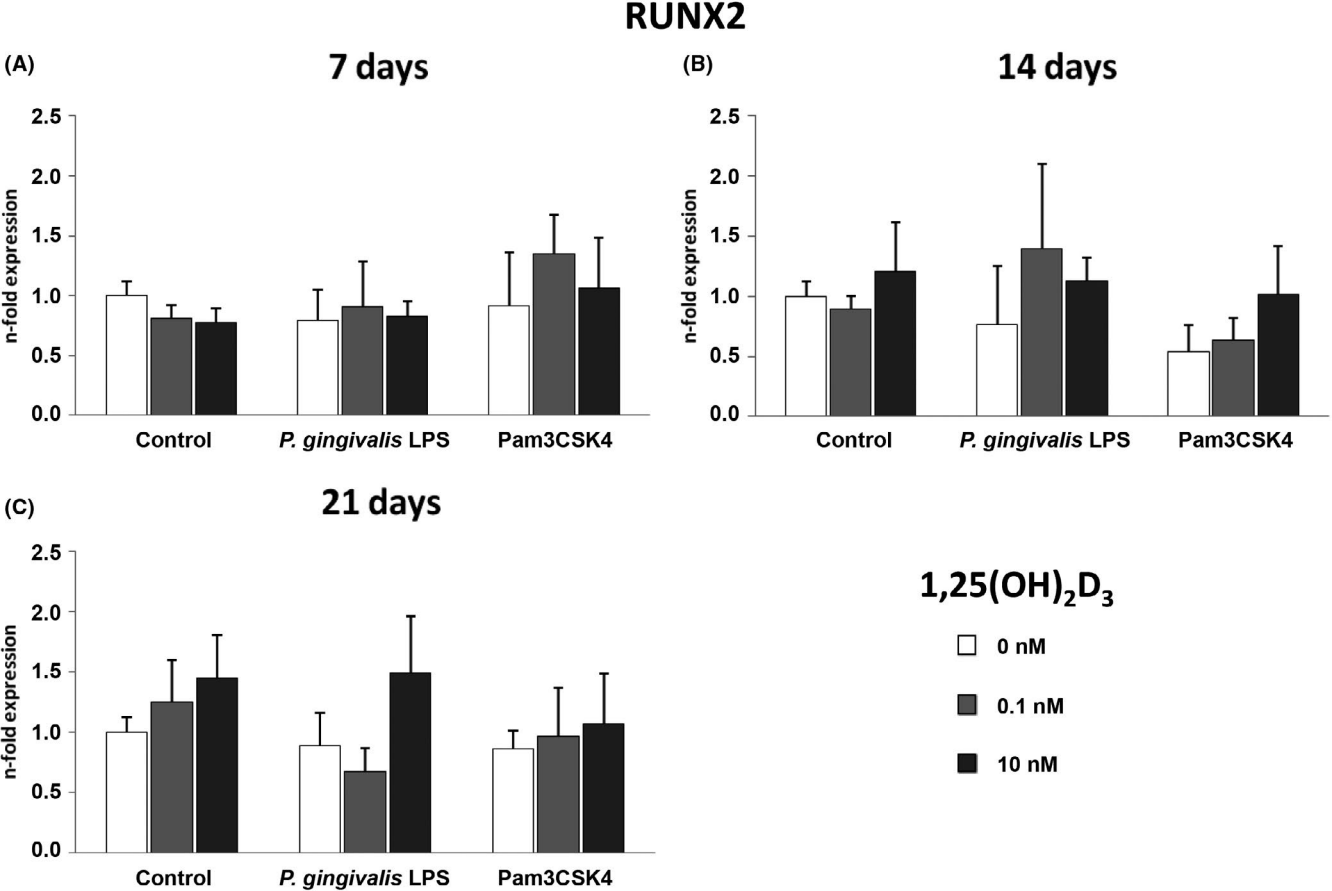
1,25(OH)_2_D_3_‐induced gene expression levels of RUNX2 under inflammatory and osteogenic conditions. Primary hPDLSCs (n = 5) were stimulated with osteogenic induction medium containing 1,25(OH)_2_D_3_ (0, 0.1, 10 nM) in the presence and absence of *P. gingivalis* LPS (1 µg/ml) or Pam3CSK4 (1 µg/ml) for 7 (A), 14 (B) or 21 (C) days. Resulting gene expression levels of RUNX2 were analysed with qPCR. Y‐axes represent the n‐fold expression of the target gene in relation to untreated hPDLSCs. Data are presented as mean ±standard error of the mean of five independent experiments

RUNX2 gene expression in hPDLSCs after 7 (A), 14 (B) and 21 (C) days culture with osteogenic induction medium containing 1,25(OH)_2_D_3_ (0, 0.1, 10 nM) in the presence and absence of 1 µg/ml *P*.* gingivalis* LPS or 1 µg/ml Pam3CSK4 is shown in Figure 3. Stimulation with different 1,25(OH)_2_D_3_ concentrations had no significant effect on the RUNX2 expression under both, physiological and inflammatory conditions.

### 3.4 Osteogenic differentiation of hPDLSCs induced by 1,25(OH)_2_D_3_ remains unaltered in the presence of inflammatory stimuli

**FIGURE 4:**
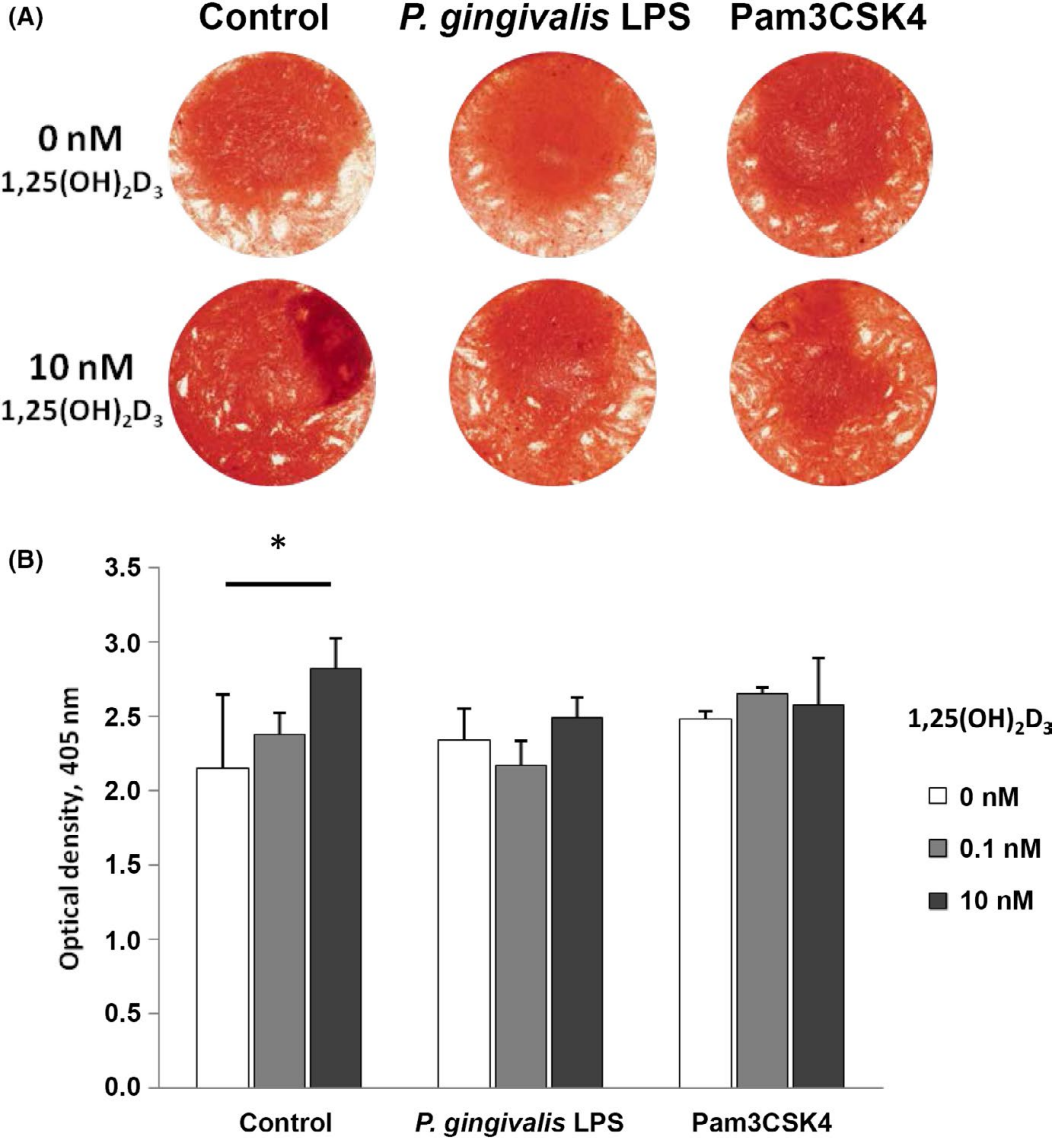
1,25(OH)_2_D_3_‐induced osteogenic differentiation under inflammatory conditions. Primary hPDLSCs (n = 5) were stimulated with osteogenic induction medium containing 1,25(OH)_2_D_3_ (0, 0.1, 10 nM) in the presence and absence of *P. gingivalis* LPS (1 µg/ml) or Pam3CSK4 (1 µg/ml) for 21 days. Resulting calcium deposition was analysed by Alizarin Red S staining (A: representative picture of one donor). Quantification of calcium deposits was performed photometrically. Y‐axis represents the optical density measured at 405 nm. Data are presented as mean ±standard error of the mean of five independent experiments. *Significant difference between groups, *p* < 0.05

Figure 4B shows the photometrical analysis of Alizarin Red S stained hPDLSCs after culture in osteogenic induction medium containing 1,25(OH)_2_D_3_ (0, 0.1, 10 nM) in the presence and absence of inflammatory stimuli *P. gingivalis* LPS (1 µg/ml) or Pam3CSK4 (1 µg/ml) for 21 days. Representative pictures of hPDLSCs (n = 1) stained with Alizarin Red S are illustrated in Figure 4A.

In the absence of inflammatory stimuli, treatment with the highest 1,25(OH)_2_D_3_ concentration resulted in a significant increase of calcium deposits. In contrast, the vitamin D_3_ metabolite had no significant effect on the optical density of Alizarin Red S stained hPDLSCs under inflammatory conditions.

### 3.5 25(OH)D_3 _has no impact on the osteogenic differentiation and expression of osteogenesis‐related factors of hPDLSCs under osteogenic conditions

**FIGURE 5:**
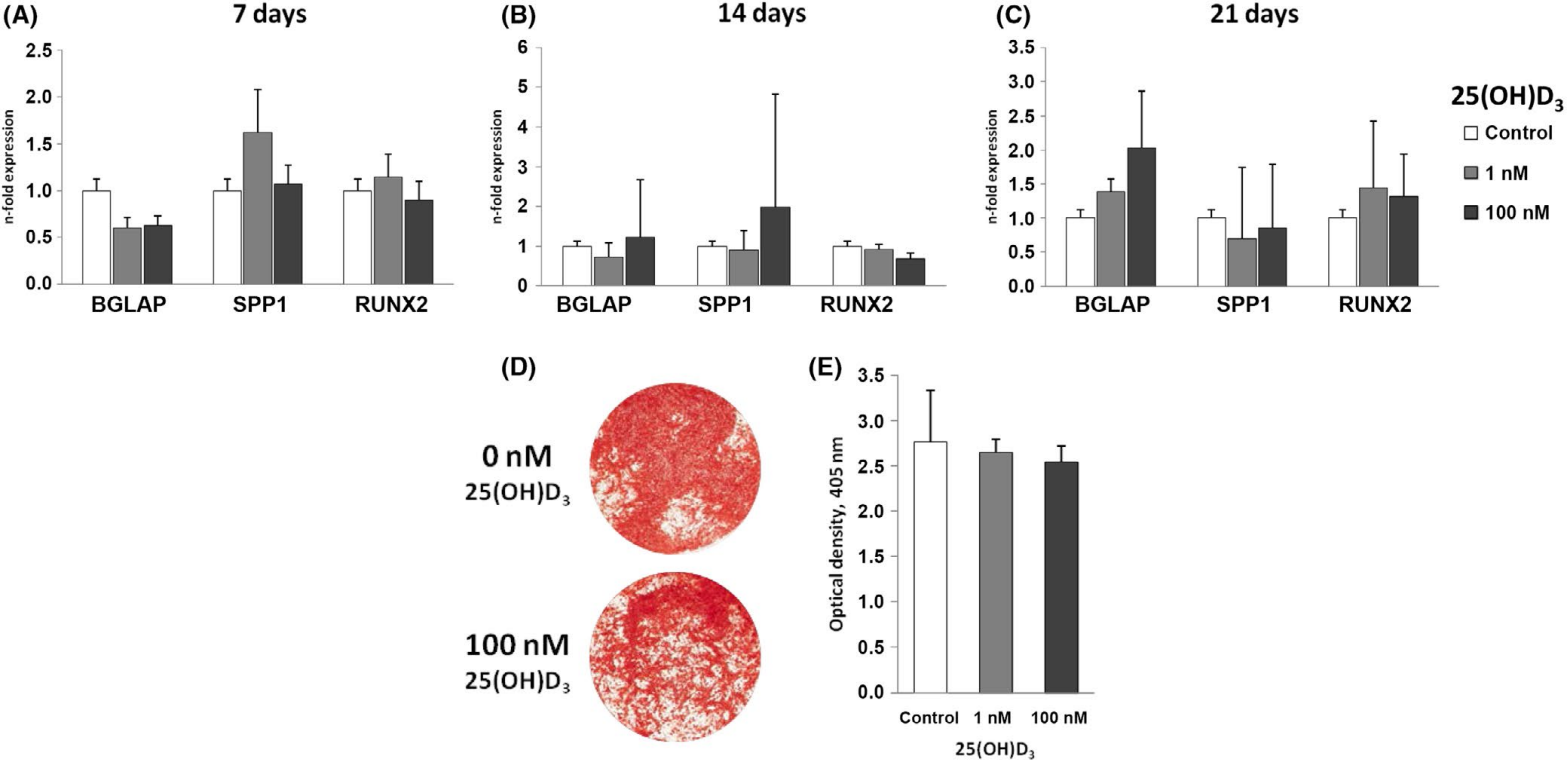
25(OH)D_3_‐induced gene expression levels of osteocalcin, osteopontin and RUNX2 and osteogenic differentiation. Primary hPDLSCs (n = 5) were stimulated with osteogenic induction medium containing 25(OH)_2_D_3_ (0, 1, 100 nM) for 7 (A), 14 (B) or 21 (C‐E) days. Resulting gene expression levels of osteocalcin, osteopontin and RUNX2 were analysed with qPCR. Y‐axes of Figure 5A, 5B and 5C represent the n‐fold expression of the target gene in relation to untreated hPDLSCs. Resulting calcium deposition was analysed by Alizarin Red S staining (D: representative picture of one donor). Quantification of calcium deposits was performed photometrically. Y‐axis of Figure 5E represents the optical density measured at 405 nm. All data are presented as mean ±standard error of the mean of five independent experiments

The gene expression levels of osteocalcin, osteopontin and RUNX2 in hPDLSCs cultured in osteogenic induction medium containing 25(OH)D_3_ (0, 1, 100 nM) for 7 (A), 14 (B) and 21 (C) days is illustrated in Figure 5. Figure 5E demonstrates the photometrical analysis of Alizarin Red S stained hPDLSCs after treatment with osteogenic induction medium containing 25(OH)D_3_ (0, 1, 100 nM) after 21 days and representative pictures are shown in Figure 5D.

25(OH)D_3_ neither had an effect on the expression of osteogenesis‐related factors after 7, 14 and 21 days, nor influenced the osteogenic differentiation of hPDLSCs. Therefore, experiments conducted under inflammatory conditions are not shown.

## 4 DISCUSSION

Apart from its fundamental role for bone metabolism, vitamin D_3_ is also well known for its anti‐inflammatory and immunomodulatory properties.^30^ Currently, it is also discussed to play a role in preventing COVID‐19 infection, progression and severity.^31^, ^32^ Vitamin D_3_ deficiency is prevalent in a pandemic extent and is associated with numerous health issues, including different bone diseases and periodontitis.^2^, ^16^, ^33^ Despite its positive effect on periodontal tissues, supplementation of vitamin D_3_ during periodontal therapy has never been shown to be beneficial so far.^34^ In an attempt to provide a possible explanation for this, our previous study investigated the effects of vitamin D_3_ metabolites on hPDLSCs in the presence of inflammatory stimuli. hPDLSCs have been shown to be strongly affected by vitamin D_3_ metabolites and are moreover able to locally convert 25(OH)D_3_ to 1,25(OH)_2_D_3._ ^6^, ^35^, ^36^ We observed that the transcriptional activity of VDR is diminished in hPDLSCs under inflammatory conditions.^22^ In particular, we could show that the 1,25(OH)_2_D_3_‐ and 25(OH)D_3_‐induced gene expression levels of osteocalcin and osteopontin were significantly decreased in the presence of *P*.* gingivalis* LPS and Pam3CSK4.

The goal of the present study was to reveal if these inflammatory stimuli also affect the vitamin D_3_‐induced osteogenic differentiation of hPDLSCs. In addition, the effects of *P. gingivalis* LPS and Pam3CSK4 on the 1,25(OH)_2_D_3_‐ and 25(OH)D_3_‐induced gene expression levels of osteogenesis‐related factors osteocalcin, osteopontin and RUNX2 were evaluated in hPDLSCs under osteogenic conditions.

In order to achieve this aim, hPDLSCs of five healthy donors were incubated in osteogenic induction medium containing different concentrations of 1,25(OH)_2_D_3_ and 25(OH)D_3_ in the presence and absence of *P. gingivalis* LPS and Pam3CSK4. Gene expression analysis of osteogenesis‐related factors was conducted after 7, 14 and 21 days of incubation, whereas osteogenic differentiation was assessed after 21 days only.

The addition of 1,25(OH)_2_D_3_ to the osteogenic induction medium significantly enhanced the osteogenic differentiation of hPDLSCs. These results are in accordance with the findings of Nebel et al and Ji et al, who assessed vitamin D_3_‐induced osteogenic differentiation via alkaline phosphatase activity and Alizarin red S staining.^14^, ^15^ Interestingly, the presence of inflammatory stimuli did not affect the 1,25(OH)_2_D_3_‐triggered osteogenic differentiation of hPDLSCs. Therefore, the attenuating effects of *P*.* gingivalis* LPS and Pam3CSK4, which have been observed in our previous study, could not be confirmed at this level.^22^ However, our results are in accordance with the recently published study of Karlis et al, who showed that TLR2 and TLR4 activation has no effect on the osteogenic differentiation of gingival fibroblasts.^37^

In order to rule out any impact of the osteogenic induction medium, the effect of inflammatory stimuli on the 1,25(OH)_2_D_3_‐triggered expression of osteogenesis‐related factors was assessed under osteogenic conditions. Similarly to the findings of our previous study, stimulation with 1,25(OH)_2_D_3_ significantly increased the osteocalcin and osteopontin levels, which was observed after all treatment periods. In the presence of Pam3CSK4, the 10 nM 1,25(OH)_2_D_3_‐induced osteocalcin expression was significantly decreased after 7, 14 and 21 days. In case of the osteopontin expression, such an effect was only observed after 14 and 21 days. In contrast to our previous study, *P*.* gingivalis* LPS did not affect the 1,25(OH)_2_D_3_‐triggered osteocalcin expression in the presence of osteogenic induction medium.^22^ However, the 1,25(OH)_2_D_3_‐induced ostepontin expression was significantly decreased by *P*.* gingivalis* LPS after 7 and 14 days.

A possible explanation for these results could be the artificial additives in the osteogenic induction medium, such as dexamethasone. This glucocorticoid increases the transcription of VDR, and thus leads to an enhanced effectiveness of 1,25(OH)_2_D_3_, which could distort the results.^38^ Moreover, dexamethasone has anti‐inflammatory properties, which could be viewed as a certain limitation for this study. However, according to Sung‐Mi et al, dexamethasone is crucial for the osteogenic differentiation of hPDLSCs and hence, its addition was inevitable.^39^

In addition, there are also other additives that might have influenced the results of this study. The additive β‐glycerophosphate has been shown to cause ectopic mineralization as long as alkaline phosphatase is present and FBS possesses considerable immunosuppressive properties and inhibits the expression of pro‐inflammatory cytokines.^40^, ^41^, ^42^, ^43^

Since all of these factors might contribute to distortions of the results, future studies should consider their potential role when studying the effect of different biologically active substances in osteogenic conditions *in vitro*. Furthermore, our data suggest that the changes in the expression of osteogenesis‐related genes are not necessarily associated with alterations in mineralization, which should be also considered by further studies.

Treatment with osteogenic medium containing 25(OH)D_3_ in a concentration similar to physiological serum levels (100 nM) had no influence on the osteogenic differentiation of hPDLSCs. We used 25(OH)D_3_ in our study, because this metabolite is the main form of vitamin D_3_ in blood, and it can be locally converted into 1,25(OH)_2_D_3_ by hPDLSCs.^36^ Several studies showed that 25(OH)D3 activates VDR‐mediated response in hPDLSCs.^6^, ^22^, ^35^ So far, only Lou and coworkers observed enhancing effects of 25(OH)D_3_ on the osteogenic differentiation of commercially available MSCs.^44^ Our recent study showed that 25(OH)D_3_ significantly enhances the expression of osteogenesis‐related proteins in hPDLSCs, but these experiments were performed in FBS‐free medium without any supplements.^22^ In contrast, in the present study we did not observe any effect of 25(OH)D_3_ on the expression of osteogenesis‐related genes in hPDLSCs cultured in osteogenic induction medium. Thus, it seems that the cellular effects of 25(OH)D_3_ in hPDLSCs depend on the experimental conditions, but the exact nature of this dependency should be further investigated.

The gene expression levels of RUNX2 were not affected by treatment with osteogenic induction medium containing vitamin D_3_ metabolites, which is in accordance with previous studies conducted on osteoblastic cell lines representing different maturation stages.^45^ Interestingly, treatment of hPDLSCs with inflammatory stimuli also had no impact on the gene expression of RUNX2. Unlike our data, Uehara et al. and Xing et al. reported inhibitory effects of *P*.* gingivalis* LPS on RUNX2 expression in hPDLSCs and bone marrow‐derived MSCs, respectively. One possible explanation of these discrepancies might be the differences in the LPS concentration, which was 10 times lower in our study.^46^, ^47^ However, the concentration used in our study has been shown to significantly enhance the inflammatory response of hPDLSCs, which was comparable to other strong inflammatory stimuli such as TLR2/1 agonist Pam3CSK4 and TLR3 agonist Poly I:C.^24^, ^25^, ^48^ Therefore, other factors such as stimulation protocol, cell source, LPS source and experimental conditions should be considered when comparing our data with those of other studies.

This study is limited by it´s *in vitro* character, which does not allow direct translation into the clinical situation. Osteogenic differentiation *in vitro* is a rather artificial process, which is hardly reflected in the *in viv*o situation. Another limitation is the use of hPDLSCs from young and healthy donors. Considering this aspect, this study could be extended by including hPDLSCs of periodontitis patients and age‐matched healthy individuals. Furthermore, since third molars experience less occlusal forces, other teeth could be utilized for cell isolation.

Summarizing the data of this study, we could show that the 1,25(OH)_2_D_3_‐triggered osteogenic differentiation of hPDLSCs is not affected under inflammatory conditions. However, the effects of 1,25(OH)_2_D_3_ seem to be, at least partially, diminished by inflammatory stimuli under osteogenic conditions. Further studies are required to reveal the influence of inflammation on the effectiveness of vitamin D_3_ metabolites.

## AUTHOR CONTRIBUTIONS

AB, CB, OA, conceived and designed the data; AB, BK, JG, acquired the data; AB, AM, XR, OA, analysed and interpreted the data; AB, OA, drafted the article; CB, BK, JG, AM, XR, critically revised and edited the study. All authors: approved the manuscript.

## Data Availability

The data that support the findings of this study are available from the corresponding author upon reasonable request.
